# Increased Cancer Incidence Following up to 15 Years after Cardiac Catheterization in Infants under One Year between 1980 and 1998—A Single Center Observational Study

**DOI:** 10.3390/jcm9020315

**Published:** 2020-01-22

**Authors:** Heiko Stern, Michael Seidenbusch, Alexander Hapfelmeier, Christian Meierhofer, Susanne Naumann, Irene Schmid, Claudia Spix, Peter Ewert

**Affiliations:** 1 German Heart Center Munich, Clinic for Pediatric Cardiology and Congenital Heart Disease, Lazarettstrasse 36, D-80636 Muenchen, Germany; meierhofer@dhm.mhn.de (C.M.); naumanns@dhm.mhn.de (S.N.); ewert@dhm.mhn.de (P.E.); 2 German Research Center for Environmental Health, Institute of Radiation Protection, Ingolstaedter Landstrasse 1, D-85764 Neuherberg, Munich, Germany; michael.seidenbusch@med.uni-muenchen.de; 3 Institute of Medical Informatics, Statistics and Epidemiology, Technical University Munich, Grillparzerstr. 18, Alexander Hapfelmeier, D-81675 Muenchen, Germany; alexander.hapfelmeier@tum.de; 4 Department of Pediatric Oncology and Hematology, Dr. von Hauner Childrens Hospital, Ludwig-Maximilians-University of Munich, Lindwurmstrasse 4, 80337 Muenchen, Germany; irene.schmid@med.uni-muenchen.de; 5 German Childhood Cancer Registry (GCCR), Institute for Medical Biostatistics, Epidemiology and Informatics, Johannes Gutenberg-University, Obere Zahlbacher Strasse 69, 55131 Mainz, Germany; clauspix@uni-mainz.de

**Keywords:** Cardiac Catheterization, children, cancer, radiation, congenital heart disease, cancer risk

## Abstract

Objective: To evaluate the incidence of cancer within the first 15 years of life in children who underwent cardiac catheterization under the age of one year. Methods: In this retrospective, single center study, 2770 infants (7.8% with trisomy 21) were studied. All infants underwent cardiac catheterization under one year of age between January 1980 and December 1998. Newly diagnosed cancer in the first 15 years of life was assessed through record linkage to the German Childhood Cancer Registry (GCCR). Cancer risk in study patients was compared to the GCCR population of children less than 15 years. Patients with trisomy 21 were compared to the Danish Cytogenic Register for trisomy 21. Effective radiation doses were calculated for each tumor patient and 60 randomly selected patients who did not develop cancer. Results: In total, 24,472.5 person-years were analyzed. Sixteen children developed cancer, while 3.64 were expected (standardized incidence ratio (SIR) = 4.4, 95% confidence interval (CI): 2.5–7.2, *p* < 0.001). There was no preferred cancer type. The observed incidence of leukemia and solid tumors in trisomy 21 was only slightly higher (1 in 476 py) than expected (1 in 609 py, *p* = 0.64). There was no direct relationship between the radiation dose and the incidence of cancer. Conclusion: Cardiac catherization in the first year of life was associated with a significantly increased cancer risk in a population with congenital heart disease.

## 1. Introduction

### 1.1. Background 

Cardiac catheterization has been an indispensable tool for diagnostic and therapeutic procedures in children with congenital and acquired heart disease over the last six decades. Although several noninvasive diagnostic alternatives such as echocardiography or magnetic resonance imaging have been developed, the number of cardiac catheterizations in children has not declined in Germany [1]. It is well known that cardiac catheterization includes a significant amount of effective radiation dose in the pediatric patient population [2,3,4,5,6,7,8]. Published doses for pediatric cardiac catheterization range from 2.2 to 12 mSv effective doses [5], but show a wide variation, depending on various factors such as type of catheter equipment or diagnostic versus interventional procedures. In particular, the child’s weight at the time of procedure is a key factor to consider [9].

Within the dose range achieved during pediatric cardiac catheterization, only stochastic biological effects will occur [10] with radiation-induced cancer being the most frequent of these stochastic effects [11]. Cancer occurs after a latency period of at least 5–10 years for most solid tumors and approximately two years for leukemia [9]. Unfortunately, children have to be considered as being much more sensitive to ionizing radiation than adults [12] as radiation dose in children under 10 years has been assumed to increase the stochastic radiation risk by a factor of 3–4 [13]. In recent years, direct epidemiologic proof has been given for increased risk of neoplastic diseases after CT examinations of patients under 22 and 19 years of age [14,15], respectively.

Clinical studies assessing the risk of cancer in children exposed to radiation during cardiac catheterization procedures are scarce [16,17] and report conflicting results. 

Conflicting results for the incidence of cancer after pediatric cardiac catheterization may originate in particular from strongly varying body weight during the first 18 years of life, resulting in a wide range of effective radiation dose. Therefore, in our study, we focused on infants under one year of life undergoing cardiac catherization. They form both a high-risk pediatric group by young age and a physically homogenous group as body weight is expected to range only between 2.5 kg (third percentile at birth) and 11.8 kg (95th percentile at one year). By this restriction on infants, physical exposure parameters become much more uniform. We hypothesized that radiation effects on cancer incidence could potentially be unmasked by excluding older children beyond one year of age.

### 1.2. Objective

The aim of this study was to evaluate the incidence of cancer in children who underwent cardiac catheterization under the age of one year in our institution between 1980 und 1998. Cancer occurrence was assessed during the first 15 years of life. The incidence was compared to the risk of cancer in the general German population as assessed annually since 1980 by the German Childhood Cancer Registry (GCCR).

## 2. Methods

### 2.1. Study Design

This is a retrospective, observational study in a tertial referral center for pediatric cardiology.

### 2.2. Participants

We included 2872 infants without known malignant disease who underwent cardiac catheterization in our institution at the age of less than one year between 1 January 1980 and 31 December 1998. The vast majority of these patients had congenital heart disease. Other indications for catherization such as myocardial biopsy for suspected myocarditis or metabolic disease, rheumatic disease or infective carditis were less than 1%. Patients had to be resident in Germany at the time of catherization. Retrieved data included date of birth, sex, date of cardiac catheter, date of last follow up in our outpatient clinic, death before the age of 15 years, diagnosis of trisomy 21, diagnosis of cancer, and date of cancer diagnosis. Trisomy 21 was retrieved because this patient group is known to present with a higher leukemia rate, compared to the general population. Data were taken from a FileMaker© (Nashoba Systems, Concord, MA, USA) data registry that had been established in our catheter laboratory, recording patients undergoing cardiac catheterization since 1974 and from our electronic patient database. In selected cases, paper records were retrieved from the clinic’s archive.

The study had been reviewed and approved by the Ethics Committee of the Technical University Munich. (Project No. 255/14). 

### 2.3. Data Sources

Patients who underwent catheterization at the age of less than one year and were documented to have been observed until the age of 14 years were cross-matched with the GCCR database for newly diagnosed cancers occurring between 1980 and 2013. The GCCR records almost all childhood cancers diagnosed until the age of 14 years in Germany since 1980 with a high degree of completeness for all cancer types. The GCCR has been used previously as a database for several childhood cancer studies in national and international reports [18,19].

Kryptographed patient data were matched to the kryptographed database of the GCCR using the software merge tool box [20].

All matches from the GCCR database were reviewed using the clinical database of our institution.

For all patients identified as having developed a malignant tumor up to an age of 14 years after catheterization in the first year of life, individual radiation exposure doses were estimated, using dose–area products that had been noted for each catherization procedure. One tumor patient was catheterized three times in the first year of life; in this patient, the cumulative radiation dose was calculated and used for analysis. As reference, dose–area products from 60 randomly selected patients, catheterized in the first year of life, who did not develop cancer until an age of 14 years, were selected. 

The analysis was restricted to the following cancer groups: all cancers, all leukemias, lymphomas, and tumors of the central nervous system (including non-malignant tumors).

**Figure 1:**
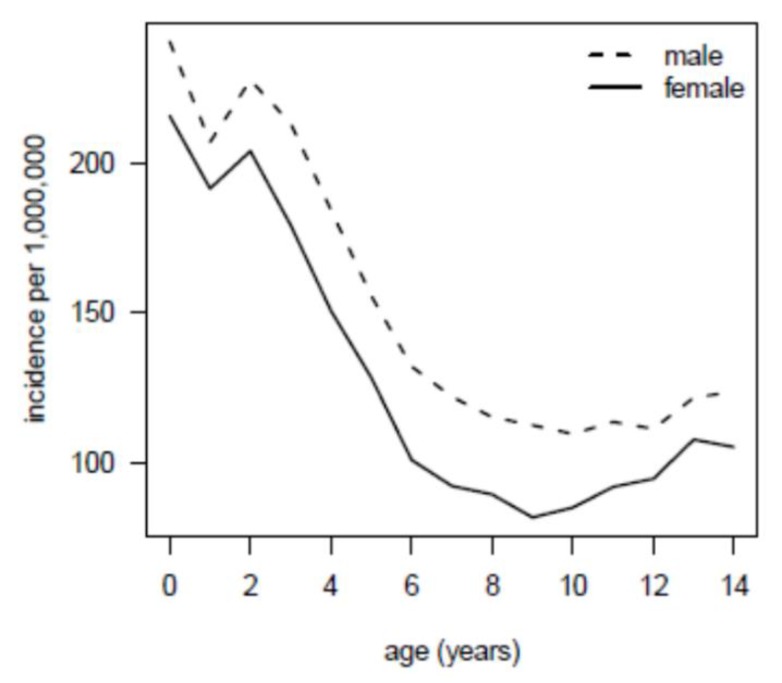
Cancer incidence of the German pediatric population between 1980 and 2014 stratified by gender and age, as reported by the German Childhood Cancer Registry.

The GCCR provided age and gender specific incidence rates of the general German population (excluding Berlin; data from East Germany were included since 1991) classified by the International Classification of Childhood Cancer [21], aggregated across the years 1980–2013 and for children with an age of 0–14 years. A corresponding illustration is given in Figure 1. 

### 2.4. Quantitative Variables-Calculation of Organ and Effective Doses

For determining a dose–effect relationship between individual radiation dose of the patient and cancer incidence, organ and effective doses were reconstructed from the cumulative dose–area products that were measured during the catheterization procedures. A dose reconstruction was performed in 16 patients having developed cancer and in 60 control patients who were randomly selected from the entire cohort and stayed free of cancer up to the age of 14 years. Effective radiation dose was estimated using dose–area products that had been registered for each catheter procedure.

For reconstruction of organ and effective doses, the commercially available PCXMC algorithm, version 2.0, was used, which was developed by the Radiation and Nuclear Safety Authority (STUK) of Finland [22,23]. The algorithm allows Monte Carlo (MC) simulations in mathematical MIRD (Medical Internal Radiation Dose Committee) phantoms of various age groups and adjustable sizes, which were constructed according to human models given by Cristy et al. [24] and the ICRP standard [25]. The phantom and the 40 reference organs contained herein are depicted elsewhere [26]. 

MC simulations were performed for each patient adjusting the MIRD phantom to the patient’s anthropometric properties (body height and body weight) considering standard sagittal and lateral field settings (cranial field edge: lower mandible; caudal field edge: 12th rib; and focus-to-skin distance: 15 cm) and standard exposure parameters (tube voltage: 63 kV; and total filtration: 3 mm aluminum) and the X-ray spectra resulting from these exposure parameters [27]. Each MC simulation was performed considering a total of 100,000 photon histories, resulting in a standard error of about 5% in organs and tissues hit by the primary radiation field. For more details, see reference [28,29].

### 2.5. Statistics

Person-years spent under the age and gender specific risks of the general population (Figure 1) were calculated for the studied cohort to infer on the expected number of cancer diagnoses. 

A chi-squared test was used for statistical hypothesis testing of this number against the actual count of observed cancer diagnoses [30]. A respective standardized incidence ratio (SIR = number of observed events/number of expected events) was computed with exact 95% confidence interval (CI) [31]. The cumulative incidence of the studied cohort was estimated by the Kaplan–Meier method and the log-log transformation was used to compute corresponding 95% confidence intervals. These were displayed in a plot against the risk of the respective general population. 

A subgroup analysis was conducted for patients with trisomy 21. Risks of a corresponding general population of children less than 15 years were taken from the Danish Cytogenic Register for trisomy 21 [32], as a German database on trisomy 21 patients does not exist. The prevalence of trisomy 21 was known for the patients who developed cancer but not for the entire study cohort. For a subgroup of 848 patients, however, it was known that 66 (7.8%) patients had trisomy 21. This prevalence of 7.8% was extrapolated to the entire cohort to infer on the total of person-years observed for this subgroup. Computations conducted for the entire cohort were repeated in the subgroup using these known cancer cases and derived person-years. 

The association between the effective radiation dose and the risk of cancer was assessed in another analysis. Doses of patients with cancer and of 60 control patients were dichotomized by an optimal cut-off value found to maximize the log-rank statistic. Hypothesis testing is based on a permutation test of this maximal test statistic [33].

All computations were performed using R 3.4.2 (R Foundation for Statistical Computing, Vienna, Austria). Statistical hypothesis was conducted on exploratory two-sided 5% significance levels.

## 3. Results

### 3.1. Participants

The catheter database included 2872 patients, who underwent cardiac catheterization in the first year of life. A total of 102 patients had to be excluded from analysis for unknown gender (*n* = 21) and missing follow-up (*n* = 81). The available data of the remaining 2770 patients (1595 male, 1175 female) were further processed before analysis. In 936 patients, in whom only the year but not the exact date of last follow-up was available, the date of last follow-up was set to 30 June. Observations were defined to start with the date of catheterization (available for 112 patients) or the date of birth. Following this conservative procedure, a total of 24,472.5 person-years could be used for analysis.

### 3.2. Main Results

This study demonstrates an increased cancer risk in children, having undergone cardiac catheterization under one year of age in our institution between 1980 and 1998. The expected number of cancer cases within 15 years of follow-up, derived from the German cancer registry, was 3.64, as opposed to the 16 observed cancer patients in our cohort (*p* < 0.001). 

Sixteen patients (nine female) were identified in the GCCR database to have developed malignant neoplasms or central nervous system tumors until the age of 14. The number of expected cancer cases was 3.64 (SIR = 4.4, 95% CI: 2.5–7.2, *p* < 0.001). Detailed tumor diagnoses of the 16 patients are listed in Table 1 along with the age at diagnosis, sex, and concomitant genetic disorder. 

**Figure 2:**
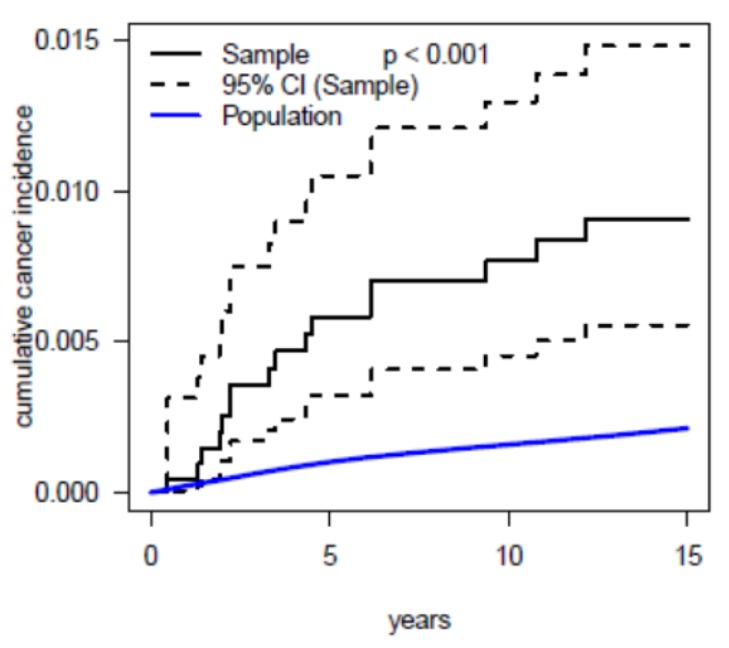
Cumulative incidence of cancer (solid black line) with 95% confidence intervals (dashed black lines) in 2770 patients having undergone cardiac catherization in the first year of life compared to expected cancer incidence (solid blue line), as derived from the German Childhood Cancer Registry.

Cumulative incidence of cancer in the studied cohort and for the corresponding general population is depicted in Figure 2.

Four out of sixteen (25%) patients with cancer had trisomy 21. Three patients had acute myeloid leukemia and one patient myelodysplastic syndrome.

This proportion was higher compared to the subcohort with known trisomy 21 status (66/848 = 7.8%, chi-squared test *p* = 0.034). In the Danish cancer registry [32] for patients with trisomy 21 at the age of 0–14, the incidence of leukemia and solid tumors is 1 in 609.3 patient-years.

The observed incidence of cancer in our patients with trisomy 21 was 1 in 476.2 patient-years (*p* = 0.64). The latter was derived from the total of 24,472.5 person-years and an estimated fraction of 66/848 patients with trisomy 21. 

### 3.3. Radiation Burden

**Figure 3:**
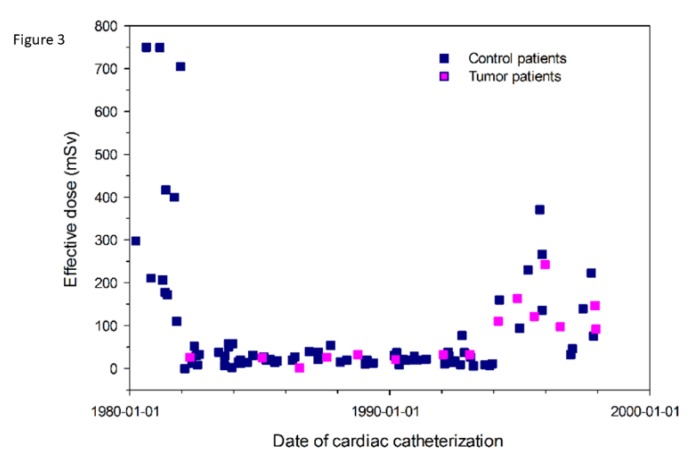
Effective radiation dose in 60 controls (blue dots) without cancer disease and 15 patients (orange dots), who developed cancer until the age of 15 years after cardiac catherization (CC) in the first year of life. Effective radiation dose is plotted against time at cardiac catherization.

Median X-ray exposition time in cancer patients was 12.3 min (range 5.0–34.9). The number of X-ray film sequences ranged 2–6 scenes in antero-posterior and lateral projections. Median effective radiation dose in 60 control patients having undergone cardiac catheterization in the first year of life, who did not develop cancer was 29.0 mSv (range: 2.0–750.0). Median effective radiation dose in 15 patients with cardiac catheter in the first year of life, who did develop cancer, was 43.0 (0.8–242.3) mSv. In one patient (Patient No. 1, Table 1), radiation dose could not be calculated due to lack of dose–area product in the paper record. Effective radiation doses in 60 control and 15 cancer patients, plotted against time at cardiac catherization, are given in Figure 3.

The hazard ratio (HR) for developing cancer was 3.6 for patients with an effective radiation dose of more than 94 mSv compared to patients with less than 94 mSv. This increased HR, however, was statistically not significant (*p* = 0.133) due to small groups of patients with known radiation burden.

## 4. Discussion

One strength of our study is that we were able to identify the last examination date and, in the case of lethal outcome, the date of death in our single center database for each patient. Therefore, the calculation of patient-years is more precise than in studies using national radiation registries [17] or cancer databases only. In such registries, death due to heart disease is usually not recorded and statistical patient-years may be overestimated. Additionally, genetic comorbidities such as trisomy 21, which may affect radiation susceptibility and cancer development, are usually not included in large radiation and cancer registries. 

Another important element of our study design is its restriction to patients under one year of age, thus improving our study’s potential to detect oncogenic effects of radiation exposure. Children have to be considered as being much more sensitive to ionizing radiation than adults [12] as radiation exposure in children under 10 years has been assumed to increase cancer risk by a factor of 3–4 [13]. Furthermore, age restriction created a more homogenous cohort in terms of body weight and physical exposure conditions. Thereby, bias through widely differing body weight and exposure conditions such as focus-to-skin-distance, filtering, and tube voltage could be minimized.

Oncogenic effects of radiation are expected to promote cancer within the first 2–10 years after exposure [9]. Cells might be damaged directly or via production of free radicals. The same ionizing radiation also has the ability to modify cells and their genetic material, thereby leading to potentially deleterious effects. The cancer incidence rate over time in our study group (Figure 2) is in line with this assumption. An increasing difference of cancer incidence between study group and general population can be observed mainly in the first 2–7 years after catherization, whereas, in the following eight years, cumulative incidence of cancer in patients is more parallel to the general population curve.

A recently published study from the Swedish Patient Register showed an overall hazard ratio for cancer of 2.24 in patients with congenital heart disease compared with healthy controls. An even higher hazard ratio of 3.37 is reported in patients born from 1990 to 1993, which fits well to our study group [34]. This study claims that radiation dose from cardiac catherization might not be the only contributor to increased risk of cancer in patients with congenital heart disease. Other factors such as lower physical activity and reduced oxygen uptake might also be reasons for increased cancer rate. This study, however, is based only on administrative, not clinical, data. Therefore, the number of patients with cardiac catherization is undetermined. The Swedish data demonstrate that, both for patients with congenital heart disease and healthy controls, the highest cancer rate occurred in the birth cohort 1990–1993. This finding is in line with our results, showing that 11 out 16 cancer cases occurred after 1990. 

Subgroup analysis revealed an increased leukemia risk in patients with trisomy 21, which had to be expected from the known increased cancer risk in trisomy 21 [32]. Generally, trisomy 21 has a 10–20-fold increased risk for leukemia, compared to the normal population [35]. Trisomy 21 was frequently observed in our study cohort, as congenital heart disease is found in approximately half of the patients with trisomy 21 [36]. Prevalence of trisomy 21 in the group of patients who developed cancer was 25%, differing significantly (*p* = 0.03) from prevalence of trisomy 21 in the whole study group (7.8%) undergoing cardiac catheterization. All cancer cases with trisomy 21 had developed leukemia or myelodysplastic syndrome. This is in line with previous data from the Danish Cytogenic Register showing that leukemia constituted 97% of malignancies in trisomy 21 in the first 15 years of life [32]. Some of the genes on chromosome 21 have been found to be disrupted in leukemia [37]. This damage might have been boosted by ionizing radiation in our cohort. 

Furthermore, one patient with Beckwith Wiedemann syndrome developed neuroblastoma. Similar to trisomy 21, Beckwith Wiedemann syndrome is considered to carry an increased risk for neoplasia, mainly Wilms tumor and hepatoblastoma but also other embryonal tumors [38]. In this patient, radiation exposure could also have been a potential trigger for neoplasia.

Clinical studies assessing the risk of cancer in children exposed to radiation during cardiac catheterizations procedures are scarce [16,17]. One Canadian study followed 4891 children who underwent at least one cardiac catheterization before the age of 18 between 1946 and 1968 and found no excess number of leukemia or solid tumors [16]. This study linked patients from the Hospital of Sick Children in Toronto, being resident in the Ontario area, with the regional Ontario Cancer Registry. Early deaths due to congenital heart disease were included, but the prevalence of trisomy 21 is unknown. Major differences to our study were older age of the study population and longer follow-up well into adulthood. Sixty-nine percent of the patients undergoing catherization were beyond one year of age and follow up was up to 35.9 years, with a median of 21.7 years. Differences in age at first catheter might account for different cancer risk compared to our study. This would confirm our hypothesis that radiation exposure in the first year of life carries a significantly higher risk for cancer compared to later years in childhood. 

A second study from Israel found an excess number of solid tumors and lymphomas in 674 children who had undergone cardiac catherization during 1950–1970 [17]. The expected number of malignancies was 4.8, while the observed number was 11.0 (SIR = 2.3). The mean age at cardiac catheter was 9.0 ± 5.6 years, meaning that only a minority of children had catherization in the first year of life. Again, this may account for the lower cancer incidence, compared to our results.

Median radiation dose tended to be higher in tumor patients, compared with a randomly selected control group without cancer during follow-up. This difference was, however, statistically not significant. Some of the effective radiation doses were high up to 700 mSv in selected patients in the early 1980s. The catheter protocol in these patients did not prove to be extraordinarily long; we speculate that this radiation burden was attributed to cardiac catheter equipment, that was not dedicated to children (no removable grid, no pediatric standard curves) in the 1980s until the mid-1990s, but had been developed for use in the adult cardiology environment. The increase in radiation dose beginning in 1994 was likely to be attributed to an increasing number of interventions. Despite high radiation doses in selected patients, cancer incidence remained to be a stochastic effect and not strictly dose related. All dose related analyses in our study were robust against these high values as they based on rank statistics.

The significantly increased cancer risk with a SIR of 4.4 (95%-CI: 2.5–7.2) in our study cohort clearly contrasts to theoretical risk calculations from previous studies, estimating a considerably lower risk [39,40]. These calculations, however, are derived from risk projection models based on studies of survivors of the atomic bombs in Japan [41]. Although these risk models attempted to account for differences between diagnostic radiation and exposure to an atomic bomb, considerable doubts remain if these calculations are correct [42].

Our data show a significantly increased cancer risk in children with congenital heart disease who had undergone cardiac catherization in the first year of life between 1980 and 1998. Technical equipment has clearly improved in the last 20 years, but the number of interventions with long study times is rising continuously in patients with congenital heart disease [43]. Therefore, the risk of inducing cancer by cardiac catherization in the young age group is still an issue. Diagnostic alternatives as catherization by use of magnetic resonance imaging (MRI) are important inputs for the reduction of ionizing radiation burden, as suggested by a recent scientific position statement [44]. In the light of a 4.4-fold increase in cancer incidence in our study, MRI as the most promising guidance of cardiac catheterization should heavily promote development of technical equipment such as wires and catheters and improve image quality of real time imaging.

### 4.1. Limitations

This study is limited to cardiac catheters in the first year of life. The number of catherizations at older age or other diagnostic X-ray procedures in the whole group is unknown. This might influence our interpretation of the results. From our data, it cannot be differentiated between cancer incidence due to radiation dose and a potentially increased cancer risk due to congenital heart disease per se. For this purpose, a second control group with congenital heart disease and without radiation exposition would have been required. Such a control group, however, was not available. Calculation of effective radiation dose is limited to cancer patients and a small number of controls. This small number limits statistical power for discrimination of tumor incidence in patients with high and low radiation burden. 

### 4.2. Conclusions

Cardiac catherization in the first year of life was associated with a significantly increased cancer risk in a population with congenital heart disease.

## Figures and Tables

**Table 1:** Patient characteristics of the 16 patients with newly diagnosed malignant tumors after having undergone cardiac catherization in the first year of life. Given are type of cancer, age at diagnosis, and concomitant genetic disorder.

Patient No	Sex	Type of Cancer	Age at Diagnosis	Genetic Disorder	Cardiac Diagnosis
1	m	Embryonal sarcoma of the lungs, metastasized	1.6	none	VSD, CoA
2	f	Acute myeloid leukemia	2.5	Trisomy 21	AVSD
3	f	Liposarcoma	12.2	none	VSD, CoA, subvalvar AS
4	m	Thyroid carcinoma	6.3	none	DILV
5	m	Lymphoid leukemia	4.5	none	VSD, PH
6	f	Neuroblastoma and ganglioneuroblastoma	3.7	none	TAPVC
7	f	Lymphoid leukemias	3.0	none	DILV, VSD, PS
8	f	Lymphoid leukemias	5.2	none	VSD, PH
9	m	intracerebral Tumor (histology unknown)	0.1	none	ASD II
10	f	Lymphoid leukemia	11.3	none	VSD, PH
11	m	Acute myeloid leukemia	1.9	Trisomy 21	ASD II
12	m	Acute myeloid leukemia	2.0	Trisomy 21	AVSD
13	f	Neuroblastoma and ganglioneuroblastoma	9.5	Beckwith Wiedemann Syndrome	Mitral stenosis, PH
14	m	Intracranial and intraspinal embryonal tumors	3.5	none	DILV, TGA
15	f	Myelodysplastic syndrome and other myeloproliferative diseases	1.9	Trisomy 21	VSD, PDA
16	f	Astrocytomas	6.2	Tuberous Sclerosis	TA, VSD, ASD

Abbreviations: VSD, ventricular septal defect; CoA, coarctation of the aorta; AS, aortic stenosis; AVSD, complete AV septal defect; DILV, double inlet left ventricle; PH, pulmonary hypertension; TAPVC, total anomalous venous connection; PS, pulmonary stenosis; ASD II, secundum atrial septal defect; TGA, transposition of the great arteries; PDA, persistent ductus arteriosus Botalli; TA, tricuspid atresia; m: male; f: female.
